# Cross talk between RNA N6‐methyladenosine methyltransferase‐like 3 and miR‐186 regulates hepatoblastoma progression through Wnt/β‐catenin signalling pathway

**DOI:** 10.1111/cpr.12768

**Published:** 2020-01-22

**Authors:** Xichun Cui, Zhifang Wang, Jianhao Li, Jianming Zhu, Zhigang Ren, Dandan Zhang, Wei Zhao, Yingzhong Fan, Da Zhang, Ranran Sun

**Affiliations:** ^1^ Pediatric Surgery Department The First Affiliated Hospital of Zhengzhou University Zhengzhou China; ^2^ Endocrinology Department The First Affiliated Hospital of Zhengzhou University Zhengzhou China; ^3^ Precision Medicine Center The First Affiliated Hospital of Zhengzhou University Zhengzhou China; ^4^ Pathology Department The First Affiliated Hospital of Zhengzhou University Zhengzhou China

**Keywords:** hepatoblastoma, METTL3, miR‐186, N6‐methyladenosine, Wnt/β‐catenin

## Abstract

**Objectives:**

N6‐methyladenosine (m6A) is a ubiquitous epigenetic RNA modification that plays a pivotal role in tumour development and metastasis. In this study, we aimed to investigate the expression profiling, clinical significance, biological function and the regulation of m6A‐related genes in hepatoblastoma (HB).

**Materials and Methods:**

The mRNA and protein expression levels of m6A‐related genes were analysed using Gene Expression Omnibus (GEO) and tissue microarray (TMA) cohort. Kaplan‐Meier analysis was performed to evaluate the prognostic value of m6A‐related genes in HB. Knockdown of m6A‐related genes was conducted to analyse its function on cell proliferation, migration and invasion. Furthermore, bioinformatics analysis and experimental verification were used to explore the potential molecular mechanism and signalling pathway.

**Results:**

We found that most m6A‐related genes were significantly upregulated in HB tumour tissues. High levels of methyltransferase‐like 3 (METTL3, *P* = .013), YTHDF2 (*P* = .037) and FTO (*P* = .032) indicated poor clinical outcomes, and the upregulation of METTL3 was an independent prognostic factor in HB patients. Functional assays showed that knockdown of METTL3 could dramatically suppress the proliferation, migration and invasion of HB cells. In addition, METTL3 was identified to be a direct target of microRNA‐186 (miR‐186). Consistently, miR‐186 was low expressed in HB tumour tissues. Moreover, overexpression of miR‐186 significantly inhibited cell aggressive phenotype both in vitro and in vivo, while the inhibitory effect could be reversed by METTL3 overexpression. Mechanism study indicated that miR‐186/METTL3 axis contributed to the progression of HB via the Wnt/β‐catenin signalling pathway.

**Conclusions:**

M6A‐related genes were frequently dysregulated in HB. miR‐186/METTL3/Wnt/β‐catenin axis might serve as novel therapeutic targets and prognostic biomarkers in HB.

## 1 INTRODUCTION

Hepatoblastoma (HB) is the most common paediatric liver malignancy, accounting for approximately 50% of paediatric hepatic‐related tumours.^1^ Though many advanced therapeutic strategies were applied, the prognosis of HB was far from satisfactory due to its late diagnosis, recurrence and metastasis.^2^ Hence, it is critical to identify novel biomarkers and develop efficient therapeutic strategies for HB patients.

N6‐methyladenosine (m6A) is the most abundant internal modification in mammalian mRNA, which has emerged in a ubiquitous role to fine‐tune RNA processing by acting as “writers,” “erasers” and “readers”.^3^ Previous studies have documented the pivotal roles of m6A‐related genes in various human diseases, through regulating RNA stability, mRNA splicing and translation, and microRNA processing.^4^, ^5^, ^6^, ^7^ Recent literatures have validated the correlation between m6A and human cancers including hepatocellular carcinoma, breast cancer, pancreatic cancer and cervical cancer.^8^, ^9^, ^10^, ^11^ However, the clinical values and biological roles of m6A‐related genes in HB have not been reported. METTL3 is an RNA methyltransferase that can modify the biological occurrence, decay and translation control of mRNA by m6A.^12^ Recent studies demonstrated that METTL3‐mediated m6A modification was involved in regulating numerous genes in various types of cancer.^12^, ^13^, ^14^ However, the expression pattern and function of METTL3 in HB are not clear.

MicroRNAs (miRNAs) are short, non‐coding RNAs that can bind to the 3′‐untranslated region (3′‐UTR) of target mRNAs to regulate gene expression, leading to the degradation of target mRNAs or translational inhibition of functional proteins.^15^, ^16^ Emerging evidence indicated that miRNAs were extensively involved in the development of multiple cancers, including HB. Various miRNAs such as miR‐17, miR‐21, miR‐19b and miR‐492 were reported to play important roles in HB.^17^, ^18^, ^19^ miR‐186 was reported to exhibit tumour‐suppressive properties in multiple cancers such as neuroblastoma, breast cancer and hepatocellular carcinoma.^20^, ^21^, ^22^ However, little is known about the expression profiling and function role of miR‐186 in HB development.

In this study, we found that m6A‐related genes were frequently dysregulated at mRNA and protein level in HB. METTL3, an m6A‐associated RNA methyltransferase, was overexpressed in HB tissues and cell lines. High level of METTL3 was associated with unpleasant prognosis of HB patients. Functional experiments elucidated that METTL3 acted as an oncogene in HB. We further demonstrated that miR‐186 directly targeted METTL3 and ectopic overexpression of miR‐186 significantly inhibited aggressive tumour phenotypes of HB, both in vitro and in vivo*.* Mechanistically, we revealed that miR‐186/METTL3 axis was critical for initiation and progression of HB by regulating Wnt/β‐catenin signalling pathway. Taken together, our findings provide innovative insights for the mechanism research and therapeutic strategies for HB treatment.

## 2 MATERIALS AND METHODS

### 2.1 GEO data sets

Two independent microarrays, including GSE75271 and GSE75283 databases, were extracted from the Gene Expression Omnibus (GEO https://www.ncbi.nlm.nih.gov/geo/). The characteristics of the data sets, such as cohort ID, RNA‐seq platform, number of samples, publication year and country, are showed in Additional file 1: Table S1.

### 2.2 TMA cohorts

The tissue microarray (TMA) containing 70 paired paraffin‐embedded HB tissues and adjacent non‐tumour tissues was obtained from the First Affiliated Hospital of Zhengzhou University (ZZU cohort). The follow‐up and clinicopathological data are listed in Additional file 2: Table S2. The study was approved by the Institutional Review Board of the First Affiliated Hospital of Zhengzhou University, and all legal guardian of children signed informed consent.

### 2.3 Cell lines and culture

The HB cell lines (HepG2, HuH‐6), hepatocellular carcinoma cell lines (HCCLM9, Hepa1‐6) and embryonic kidney cell lines (HEK293) were purchased from the Cell Bank of the Chinese Academy of Science (Shanghai, China). Normal liver cell lines Chang liver and L02 were obtained from American Type Culture Collection (ATCC) or Shanghai Institute of Biochemistry and Cell Biology (SIBCB; Shanghai, China), respectively. Cells were cultured in Dulbecco's modified Eagle's medium (DMEM) supplemented with 10% foetal bovine serum and 100 U/mL penicillin/streptomycin (Corning, NY, USA) in a conventional incubator (5% carbon dioxide, 95% air) at 37°C. The details of these cells are shown in Additional file 3: Table S3.

### 2.4 Immunohistochemistry staining

Immunohistochemistry staining (IHC) of m6A‐related genes was performed according to the manufacturer's instructions.^23^ In accordance with different staining intensity and the proportion of positive cells, we established a semi‐quantitatively scoring system, and the proportion of positive cells were scored as follows: 0, none; 1+, <25%; 2+, 25%‐50%; 3+, 50%‐75%; and 4+, 75%‐100%. The staining intensity was scored as follows: 0, none; 1+, weak; 2+, medium; and 3+, strong. Total score was calculated by multiplying two subscores, and the samples with scores of 0‐6 were deemed as low expression and 7‐12 scores were classified as high expression. Two independent pathologists who were blinded to the clinical data accomplished categorizing of the m6A‐related gene staining. Additional file 4: Table S4 listed the antibody information used in this study.

### 2.5 Transfection

METTL3 siRNA (si‐METTL3), METTL3 overexpression plasmid (METTL3), miR‐186 mimics (miR‐186), miR‐186 inhibitor (anti‐miR‐186) and their corresponding negative control (NC) were obtained from GenePharma. Transfection was performed using Lipofectamine 2000 (Thermo Fisher) following the manufacturer's protocols. The expression levels of METTL3 or miR‐186 after transfection were analysed by qPCR and/or Western blotting 48‐72 hours later.

### 2.6 Western blot

RIPA buffer was utilized to extract total protein from cultured cells. Following extraction, BCA assays (Beyotime) were performed to quantify all proteins. Equal amount of protein samples was separated by 12% SDS‐PAGE and then transferred to the nitrocellulose membranes (Millipore). The membranes were blocked with 5% non‐fat milk/PBS for 1 hour. Then, the membranes were incubated by primary antibodies at 4°C overnight. After washing the membranes with PBST for three times, the membranes were further incubated with secondary antibodies for 2 hours. The membranes were developed using enhanced chemiluminescence solution (Beyotime) and exposed to the photographic film for visualization. Additional file 4: Table S4 listed the information of antibodies.

### 2.7 Real‐time quantitative PCR (RT‐qPCR)

Total RNA was extracted utilizing TRIzol reagent (Life Technologies). TransScript First‐Strand cDNA Synthesis SuperMix (TransGen) was used to reverse‐transcribe cDNA. RT‐qPCR assay was performed using PowerUp SYBR Green Kit (ABI) and QuantStudio 6 System (ABI). Data were analysed using the comparative Ct method (2^−ΔΔ^Ct). β‐Actin was served as the internal control.

### 2.8 Cell proliferation assay

Cell growth was evaluated using CCK‐8 kit (Beyotime). The DNA synthesis rate was evaluated using EdU assay kit (Ribobio). EDU‐stained cells (red fluorescence) and DAPI‐stained cells (blue fluorescence) were used to evaluate cell proliferation activity. For colony formation assay, transfected cells were seeded in 6‐well plates and cultured for 2 weeks. Then, cells were fixed with 30% formaldehyde for 15 minutes and stained with 0.1% crystal violet. The number of colonies (defined as containing more than 50 cells) was determined under a light microscopy.

### 2.9 In vitro migration and invasion assays

Wound‐healing assay was conducted to determine cell migration activity. HepG2 and HuH‐6 cells (5 × 10^6^) were seeded into six‐well plates. A 1‐mm‐wide wound was scratched when 80% cell confluence was reached. The wounded areas were washed with PBS and photographed at 0 and 48 hours. Cell invasion assay was conducted using Transwell Matrigel chambers coated with Matrigel (BD Biosciences). Upper chambers were seeded with 1 × 10^4^ transfected cells in serum‐free media, and the bottom chamber contained DMEM supplemented with 10% FBS. 24 hours later, the invasive cells were fixed, stained, photographed and quantified.

### 2.10 Luciferase assays

Wild‐type and mutated 3′‐UTR fragments of METTL3 mRNA containing the predicted miR‐186‐binding site were amplified and cloned into the psiCHECK‐2 luciferase reporter vector (Promega). HEK293 cells were co‐transfected with miR‐186 mimics or NC control, together with either METTL3‐wt or METTL3‐mut reporter vectors. After 48 hours, the Dual‐Luciferase Reporter Assay System (Promega) was used to determine the relative luciferase activity.

### 2.11 Lentiviral transduction and vector construction

The coding sequences of human miR‐186 or METTL3 were amplified and cloned into pcDNA3.1 (+; GenePharma) to generate overexpression vectors. An empty pcDNA3.1 vector was used as the negative control. HuH‐6, Hepa1‐6 and HCCLM9 cells (1 × 10^5^) were transduced with lentivirus encoding miR‐186 mimics, miR‐186 inhibitor, METTL3 overexpression plasmid, METTL3 shRNA or an empty lentivirus for 96 hours and then selected by puromycin treatment (Santa Cruz Biotechnology) for 4 weeks.

### 2.12 Tumour xenografts

Animal experiments were approved by the Animal Health Committee of Zhengzhou University. A total of 60 nude mice (male, 4‐6 weeks old) and 12 C57BL/6 mice (male, 8 weeks old) were obtained from Beijing Vital River Laboratory Animal Technology. Cells transfected with miR‐186 overexpression lentivirus (Lenti‐miR‐186), miR‐186 inhibitor (Lenti‐anti‐miR‐186), Lenti‐miR‐186 & METTL3 overexpression plasmid (Lenti‐METTL3), Lenti‐anti‐miR‐186 & METTL3 shRNA (sh‐METTL3) or empty lentivirus control (Lenti‐NC) were subcutaneously injected into the lower flank of nude mice. Tumour growth was monitored for 5 weeks. HuH‐6 cell lines were selected to establish xenograft model in nude mice. Liver orthotopic transplantation model was established with Hepa1‐6 cells. HCCLM9 cells were used to establish lung metastasis model.

After 2 weeks, a mouse model of orthotopic transplantation of the liver was established by collecting the subcutaneous tumour derived from Hepa1‐6 cells and transplanting 1‐mm^3^ samples into the liver left lobe of the C57BL/6 mouse. Tumour growth was examined every week. Mice were euthanized at 5 weeks post‐orthotopic transplantation. Photographs were recorded using the IVIS Lumina II System (Caliper Life Sciences). Tumour tissues were extracted and processed for further IHC staining.

Lung metastasis model was established by HCCLM9 cell lines. The mice in different groups received a tail vein injection of pre‐treated HCCLM9 cells. All mice were sacrificed 5 weeks after the injection. Lung tissues were also sectioned and processed for haematoxylin‐eosin (HE) staining to determine lung metastasis.

### 2.13 Statistical analysis

Statistical analysis was performed by spss 23.0 (SPSS Inc) and graphpad prism 7.0. The Student *t* test was used to analyse the difference between two groups. Clinicopathological characteristics in HB were analysed by the chi‐square tests. Overall survival (OS) of HB patients was calculated with Kaplan‐Meier curves and log‐rank tests. Univariate and multivariate Cox regression analyses were performed to identify the independent prognostic factors. The correlation was performed by Spearman rank analysis with graphpad prism 7.0. A P‐value of < .05 was considered statistically significant.

## 3 RESULTS

### 3.1 M6A “writers” METTL3 is highly expressed in HB and associated with poor prognosis

**Figure 1:**
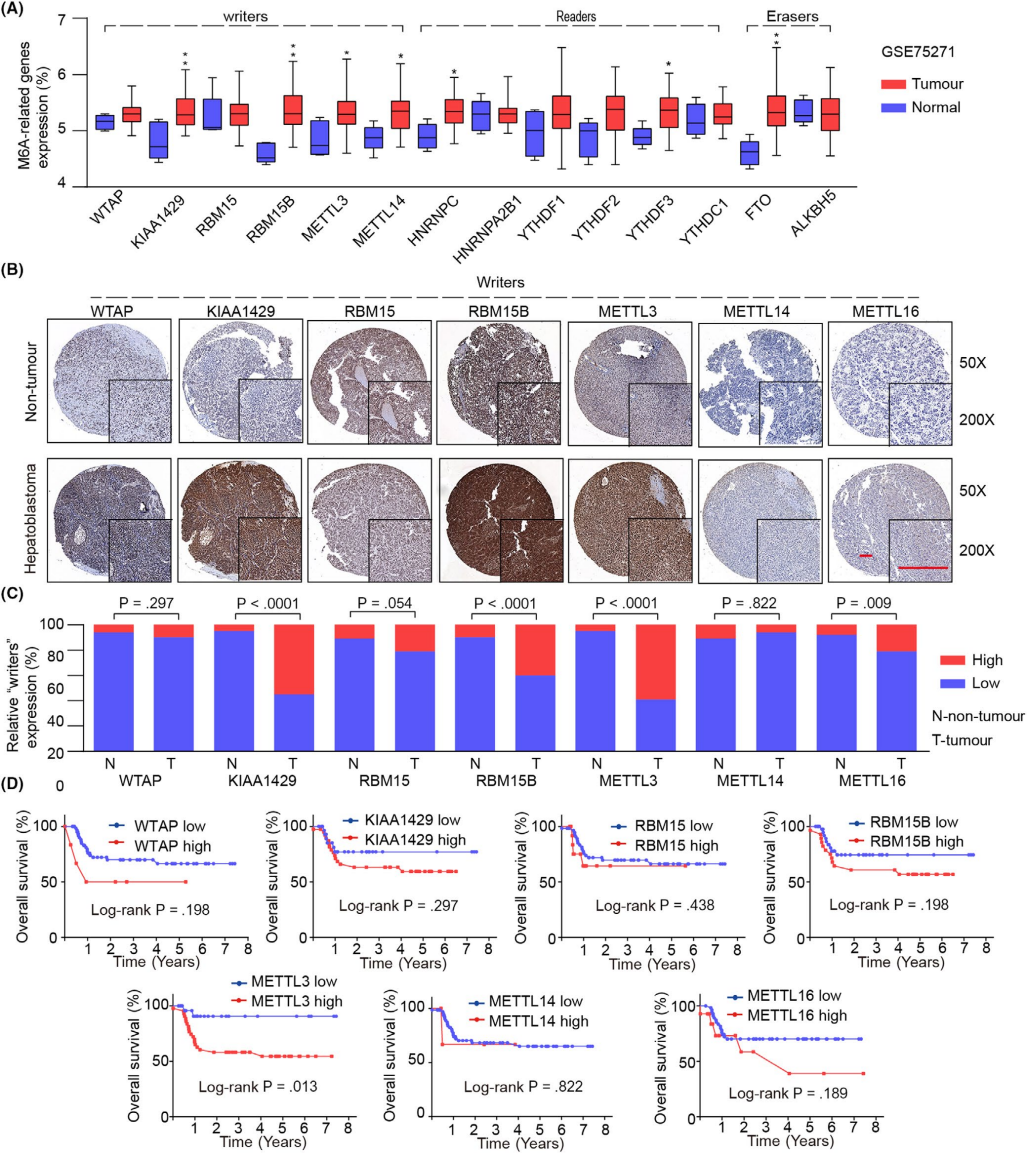
The expression patterns of m6A‐related “writers” and their association with prognosis in HB patients. A, The mRNA expression profiling of m6A‐related genes in GEO cohort (GSE75271). B, Representative IHC staining of m6A‐related “writers” in TMA (ZZU cohort) of HB tissues and normal non‐tumour tissues. Scale bars, 100 μm. C, Comparison of m6A‐related “writers” relative expression between HB tissues (T) and normal tissues (N) in HB TMA (ZZU cohort). D, Kaplan‐Meier analysis of the correlation between m6A “writers” expression and overall survival of HB patients in ZZU cohort. *P < .05, **P < .01

We first explored the expression pattern of m6A‐related genes in GEO data set (GSE75271; Figure 1A). M6A‐related genes can be categorized into “readers,” “writers” and “erasers”.^24^ We found that almost all m6A “writers” were dramatically upregulated in HB tissues compared with those in normal tissues, with the exemption of METTL16 that was absent in GEO data set. Considering the difference between the mRNA and protein expression levels of m6A‐related genes, IHC staining of tissue microarray (TMA, ZZU cohort) containing 70 paired paraffin‐embedded HB specimens was analysed to further validate the protein levels of the m6A “writers” in HB (Figure 1B). Based on the proportion of positive cells and the different staining intensity by IHC analysis, we divided the m6A “writers” expression into high and low groups. The protein expression levels of most m6A “writers” were in accordance with their mRNA levels, with the exemption of METTL14 that had a weak expression at the protein level (Figure 1C).

To further investigate the prognostic roles of m6A “writers” in progression of HB, we analysed HB patients’ overall survival data with corresponding clinical follow‐up information using the ZZU cohort (Figure 1D). Kaplan‐Meier analysis and a log‐rank test uncovered that patients with high METTL3 expression had significant worse overall survival in comparison with that in patients with low METTL3 expression (Figure 1D).

### 3.2 M6A “readers” and “erasers” expression patterns in HB and their association with prognosis in HB patients

**Figure 2:**
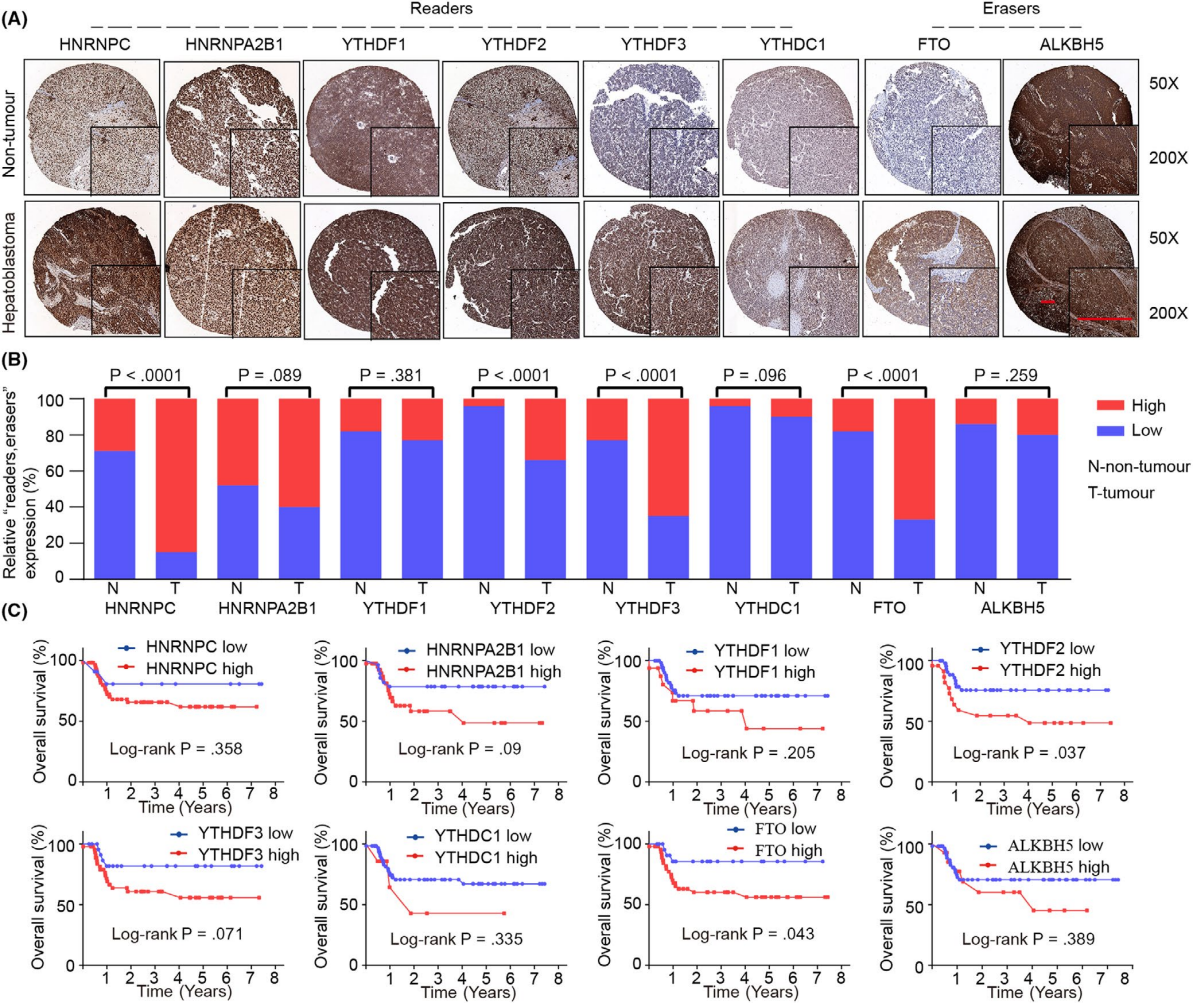
M6A “readers” and “erasers” expression patterns in HB and their association with prognosis in HB patients. A, Representative IHC staining of m6A‐related “readers” and “erasers” in TMA (ZZU cohort) of HB tissues and normal non‐tumour tissues. Scale bars, 100 μm. B, Comparison of relative expression of m6A‐related “readers” and “erasers” between HB tissues (T) and normal tissues (N) in HB TMA (ZZU cohort). C, Kaplan‐Meier analysis of the correlation between m6A “readers” or “erasers” expression and overall survival of HB patients in ZZU cohort

We further analysed the mRNA and protein expression of m6A “readers” and “erasers” through GEO database analysis and IHC staining of TMA (ZZU cohort; Figures 1A and 2A). We observed that the protein expression levels of m6A “readers” and “erasers” were in accordance with their mRNA levels, with significant higher HNRNPC, YTHDF2, YTHDF3 and FTO in HB (Figure 2B). To evaluate the potential contribution of m6A “readers” and “erasers” in HB progression, we analysed HB patients’ overall survival data of ZZU cohort (Figure 2C). We demonstrated that increased expressions of YTHDF2 (*P* = .037) and FTO (*P* = .043) were significantly associated with poor overall survival rates in HB patients.

### 3.3 High expression level of METTL3 is correlated with poor prognosis in HB

**Figure 3:**
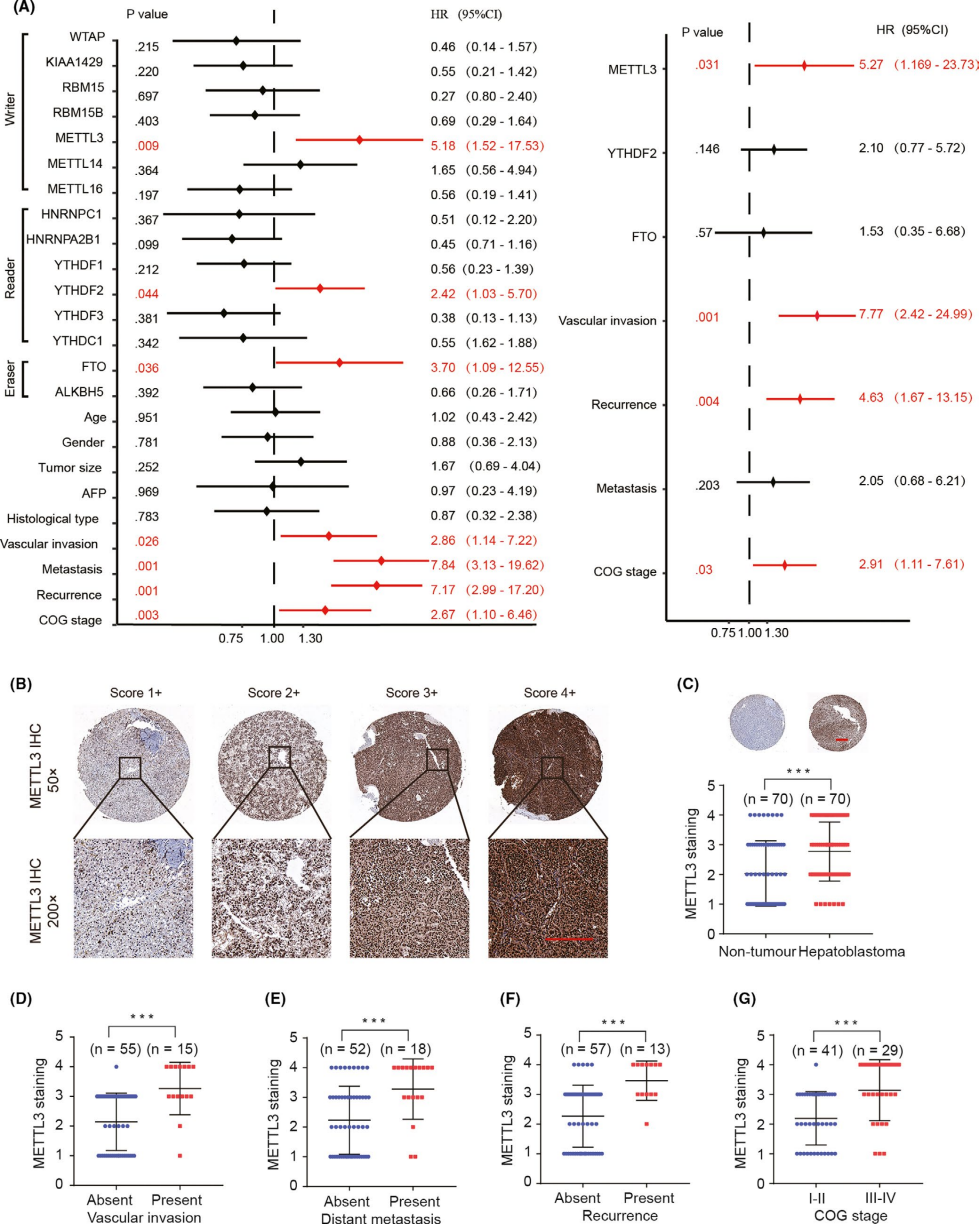
High expression level of METTL3 is correlated with poor prognosis in HB. A, The univariate and multivariate Cox regression analyses were performed to depict the correlations between the indicated clinical criteria and the expression levels of m6A‐related genes. B, C, Representative IHC staining and scoring of METTL3 in HB tissues and normal non‐tumour tissues of TMA (ZZU cohort). Scale bars, 200 μm. D‐G, The correlation between the expression of METTL3 and vascular invasion (D), distant metastasis (E), recurrence (F) and COG stage (G). ***P < .001

To corroborate the relationship between the expression of m6A‐related genes and prognosis in HB patients, univariate and multivariate Cox regression analyses were performed. The results showed that vascular invasion, metastasis, recurrence, COG stage and high METTL3 expression were independent risk factors for survival in children with HB (Figure 3A, Additional file 5: Table S5). In addition, METTL3 protein expression was assessed in HB TMA by IHC (Figure 3B). We confirmed that the expression of METTL3 was significantly upregulated in HB tissues (Figure 3C). Moreover, clinicopathological analysis demonstrated that patients with high METTL3 expression exhibited more advanced clinical features (such as vascular invasion, distant metastasis, recurrence and COG stage) than those with low METTL3 expression (Figure 3D‐G).

### 3.4 Knockdown of METTL3 inhibits proliferation, migration and invasion of HB cells in vitro

**Figure 4:**
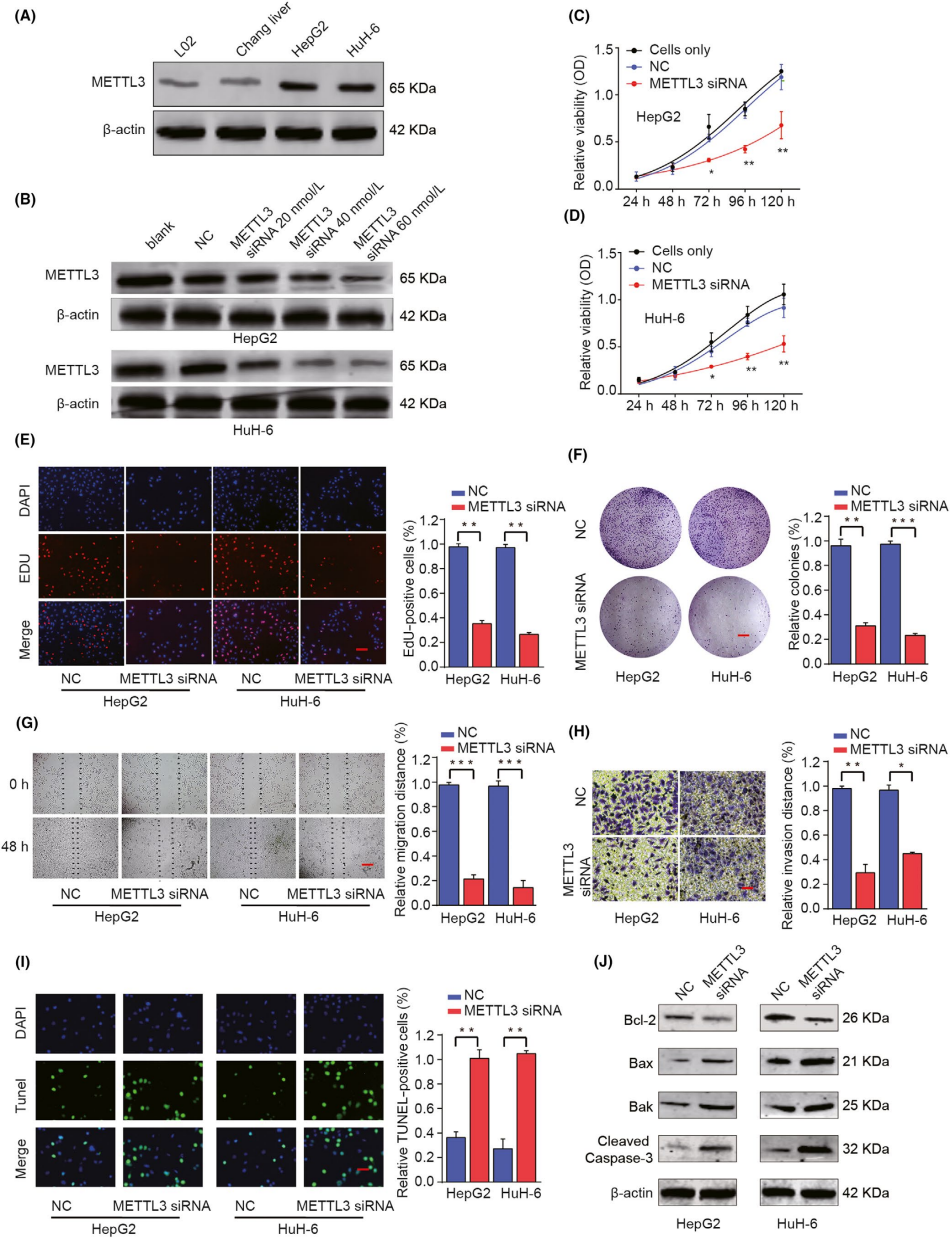
Knockdown of METTL3 inhibits proliferation, migration and invasion of HB cells in vitro. A, The protein expression levels of METTL3 in HB cell lines (HepG2 and HuH‐6) and normal liver cell lines (L02 and Chang liver) were determined by Western blot analysis. HepG2 or HuH‐6 cells were transfected with negative control (NC) or different concentrations of METTL3 siRNA. B, The protein expression levels of METTL3 were analysed by Western blot 48 h later. C, D, CCK‐8 assay, (E) EDU staining (scale bars, 50 μm) and (F) colony formation assays (scale bars, 8 mm) were performed to determine the cell proliferation, DNA synthesis and colony formation in HepG2 or HuH‐6 cells. G, Wound‐healing assay (scale bars, 500 μm) and (H) Transwell assay (scale bars, 50 μm) were performed to assess the cell migration and invasion capacity. I, Representative micrographs and quantification of TUNEL‐positive signalling in the indicated assay (scale bars, 50 μm). J, Western blot analysis of Bcl‐2, Bax, Bak and cleaved caspase‐3 proteins in HepG2 and HuH‐6 cells. *P < .05, **P < .01, ***P < .001

We further validated the expression pattern of METTL3 in HB cell lines and found higher expression levels of METTL3 in HepG2 and Huh‐6 cell lines compared with that in normal liver cell lines (L02 and Chang liver; Figure 4A). To explore the function of METTL3, siRNAs targeting METTL3 were transfected into HepG2 and HuH‐6 cells and the knockdown efficiency was evaluated (Figure 4B). CCK‐8, EDU staining and colony formation assays revealed that knockdown of METTL3 dramatically inhibited HB cell proliferation, DNA synthesis and colony formation in comparison with that in HB cells transfected with NC control (Figure 4C‐F). Silencing METTL3 could also inhibit the migration and invasion capacity of HB cells (Figure 4G,H). Furthermore, TUNEL staining assay indicated that the percentage of apoptotic cells in METTL3‐knockdown HB cells was much higher than that in control cells (Figure 4I). In addition, we observed a decrease in the anti‐apoptotic Bcl‐2 and an increase in the pro‐apoptotic Bax and Bak proteins, with an increased level of cleaved caspase‐3 in cells transfected with METTL3 siRNA (Figure 4J and Additional file 6: Figure S1). These above results suggested that METTL3 promotes the proliferation and invasion of HB cells.

### 3.5 miR‐186 targets METTL3 and negatively regulates METTL3 expression

**Figure 5:**
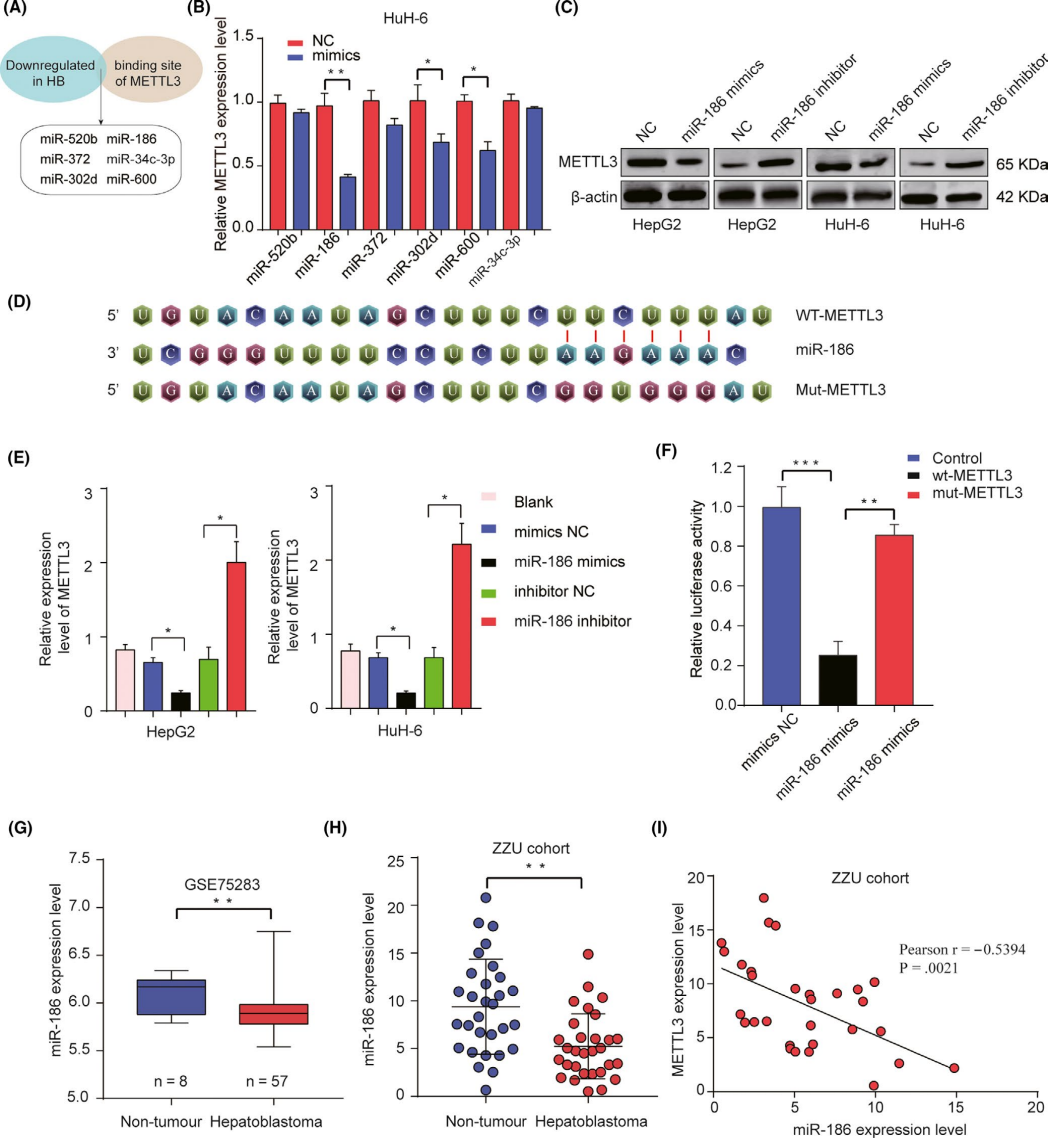
miR‐186 directly targets METTL3 and negatively regulates METTL3 expression. A, A total of 6 miRNA (miR‐520b, miR‐186, miR‐372, miR‐302d, miR‐600 and miR‐34c‐3p)‐targeted METTL3 were selected. B, A total of 6 miRNAs (miR‐520b, miR‐186, miR‐372, miR‐302d, miR‐600 and miR‐34c‐3p) were transfected into HuH‐6 cells, and the inhibitory effect on METTL3 expression was evaluated by qRT‐PCR. C, The protein expression levels of METTL3 were assessed by Western blot. D, Bioinformatics analysis predicted miR‐186 binding sites located in the 3’‑UTR of METTL3. E, The mRNA expression levels of METTL3 were assessed by qRT‐PCR. F, WT 3’‐UTR or mutated 3’‐UTR of METTL3 was cloned and constructed into luciferase reporter vector, and then co‐transfected with miR‐186 mimics or NC control into HEK293 cells. Relative luciferase activity was assessed 48 h after transfection. G, The expression of miR‐186 in HB tissues and non‐tumour tissues in GEO database was analysed. H, The expression of miR‐186 in HB tissues and non‐tumour tissues in ZZU cohort was assessed by qRT‐PCR. I, Pearson correlation analysis of the relationship between METTL3 expression level and miR‐186 expression level in ZZU cohort. *P < .05, **P < .01, ***P < .001

To explore the potential target miRNAs of METTL3 in HB, bioinformatics analysis was performed by using several online databases, including TargetScan, miRanda, miRbase and miRGen. We reasoned that candidate miRNA would be: (a) downregulated in HB tissues, and (b) with potential binding sequence for 3’‐UTR of METTL3. Based on these criteria, a total of 6 miRNAs (miR‐520b, miR‐186, miR‐372, miR‐302d, miR‐600 and miR‐34c‐3p) were chosen for further validation (Figure 5A). These candidate miRNAs were transfected into HuH‐6 cells, and the inhibitory effect on METTL3 expression was evaluated after 48 hours (Figure 5B). We found miR‐186 exhibited the most efficient suppression of METTL3 expression. Thus, we chose miR‐186 for further studies. HepG2 and HuH‐6 cells were transfected with miR‐186 mimics, miR‐186 inhibitor and corresponding NC. The results showed that there was a strong negative correlation between the protein expression levels of miR‐186 and METTL3 (Figure 5C). Meanwhile, miR‐186 was predicted to directly bind to 3’‐UTR of METTL3 (Figure 5D). Moreover, miR‐186 mimics inhibited METT3 expression, HepG2 or HuH‐6 cells treated with miR‐186 inhibitor markedly enhanced METTL3 expression (Figure 5E). Luciferase reporter assay was performed to validate that METTL3 was a direct target gene of miR‐186 (Figure 5F). Furthermore, the analysis of GEO data set (GSE75283) showed miR‐186 was significantly downregulated in HB tumour tissues, which was also validated in our ZZU cohort by qRT‐PCR analysis (Figure 5G,H). In addition, Pearson correlation coefficient analysis demonstrated that there was a significant negative correlation among miR‐186 and METTL3 expression (Figure 5I). In conclusion, these results indicate that METTL3 is a direct target of miR‐186.

### 3.6 Overexpression of miR‐186 inhibits HB cell proliferation, migration and invasion through targeting of METTL3

**Figure 6:**
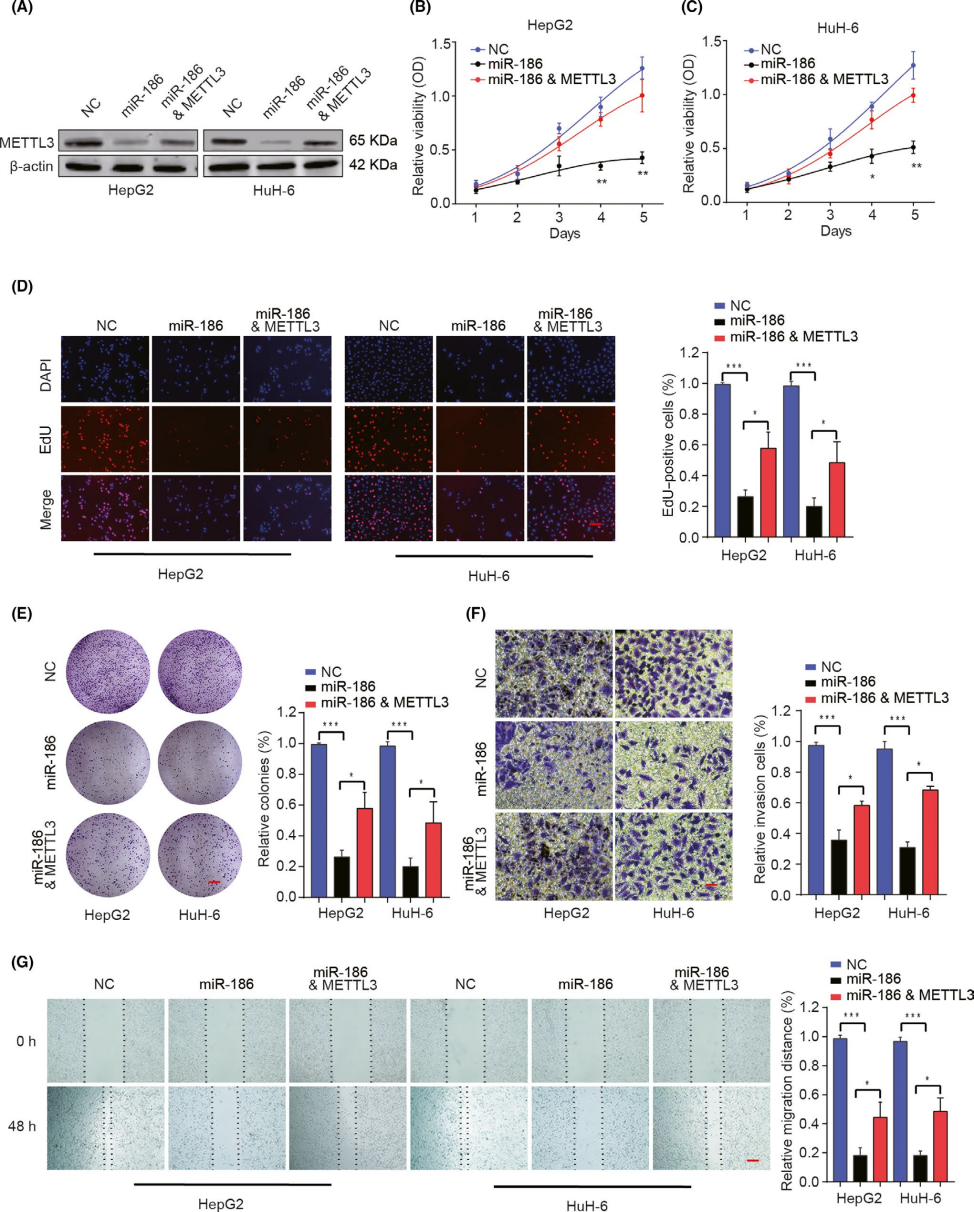
Overexpression of miR‐186 suppresses HB cell proliferation and invasion by targeting of METTL3. HepG2 or HuH‐6 cells were transfected with NC, miR‐186 mimics, and miR‐186 mimics & METTL3 overexpression plasmid. A, Western blot analysis of METTL3 protein levels in different groups. B, C, CCK‐8 assay, (D) EDU staining (scale bars, 50 μm) and (E) colony formation assays (scale bars, 8 mm) were performed to evaluate cell proliferation, DNA synthesis and colony formation of HepG2 or HuH‐6 cells. F, Cell invasion of HepG2 or HuH‐6 cells in different groups was analysed by Transwell assay. Scale bars, 50 μm (G) Cell migration capability of HepG2 or HuH‐6 cells in different groups was analysed by wound‐healing assay. Scale bars, 500 μm. *P < .05. **P < .01, ***P < .001

To further investigate the function of miR‐186 in HB cells, miRNA negative mimics (NC), miR‐186 mimics (miR‐186), or miR‐186 mimics & METTL3 overexpression plasmid (miR‐186 & METTL3) were transfected into HepG2 or HuH‐6 cells. miR‐186 overexpression significantly inhibited METTL3 expression, while overexpression miR‐186 together with METTL3 partially restored the expression of METTL3 (Figure 6A). CCK‐8 assay, EDU staining and colony formation experiments demonstrated that miR‐186 overexpression significantly inhibited proliferation, DNA synthesis and colony formation both in HepG2 and in HuH‐6 cells, but the inhibitory effect could be partially reversed by overexpression of METTL3 (Figure 6B‐E). Consistently, overexpression METTL3 could also partially reverse the inhibitory effect of miR‐186 on migration and invasion of HB cells (Figure 6F,G). On the contrary, the inhibition of miR‐186 could significantly promote HB cell proliferation, migration and invasion compared with those in the NC group (Additional file 7: Figure S2). In conclusion, all these data indicate that ectopic overexpression of miR‐186 inhibits HB cell mobility by targeting METTL3.

### 3.7 miR‐186 suppresses tumour growth and metastasis in vivo

To investigate whether miR‐186 could regulate tumour development in vivo, HuH‐6, Hepa1‐6 and HCCLM9 cells were transfected with different lentivirus vectors and cells stably overexpressing miR‐186, overexpressing METTL3, silencing METTL3 and knockdown miR‐186.

**Figure 7:**
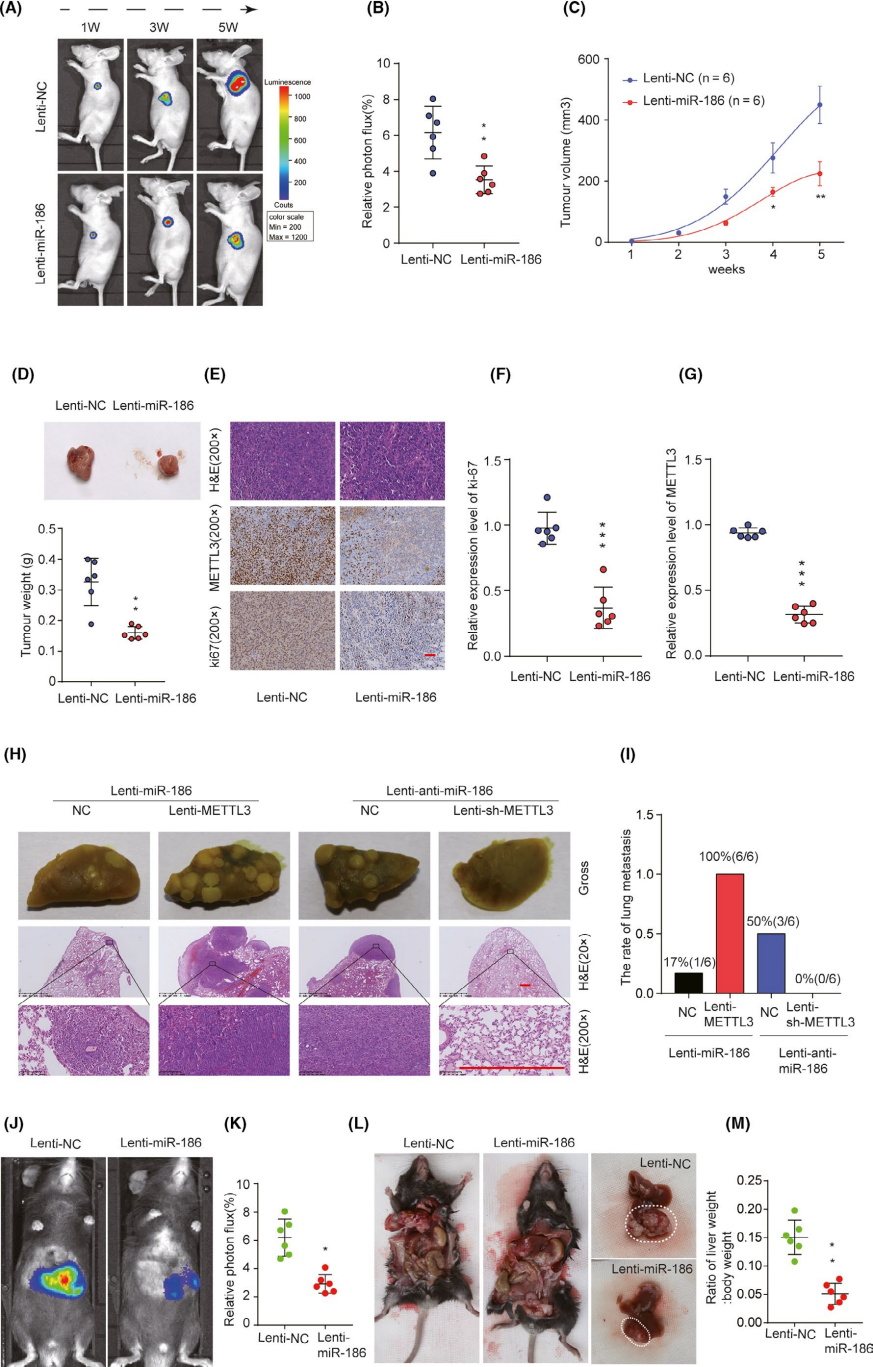
miR‐186 suppresses tumour growth and metastasis in vivo*.* A, Representative image of Luciferase signal emission in Lenti‐miR‐186 group and Lenti‐NC group. B, Relative photon flux in the Lenti‐miR‐186 group and Lenti‐NC group was quantified and analysed using the IVIS imaging system 5 wk after implantation. C, Growth curves of tumour volumes in xenografts of nude mice were determined based on tumour volume measured every week. D, Representative images of tumour and quantitative analysis of xenograft tumour weight in Lenti‐NC and Lenti‐miR‐186 groups. E‐G, Representative immunohistochemical staining images and relative expression levels of Ki‐67 and METTL3 in tumours from Lenti‐miR‐186 group and Lenti‐NC group. Scale bars, 200 μm. H, I, Representative results of gross and H&E staining of metastatic lung nodules in different groups. Scale bars, 200 μm. J, K, Relative photon flux of orthotopic transplanted tumour in Lenti‐miR‐186 and Lenti‐NC groups was quantified and analysed. L, Representative images of orthotopic tumours in the liver of Lenti‐miR‐186 and Lenti‐NC groups after dissection. M, The ratio of liver weight/body weight in orthotopical mice from Lenti‐miR‐186 and Lenti‐NC groups.*P < .05, **P < .01, ***P < .001

As shown in Figure 7A‐C, overexpression of miR‐186 significantly inhibited tumour growth as shown by luciferase photon flux and tumour volume at different time points after implantation. A significant decrease in tumour weight was observed in the miR‐186 overexpression group (Figure 7D). IHC staining of Ki‐67 and METTL3 also showed decreased expression of Ki‐67 and METTL3 in the Lenti‐miR‐186 group (Figure 7E‐G). On the contrary, inhibition of miR‐186 could significantly promote tumour growth compared with that in the Lenti‐NC group (Additional file 8: Figure S3).

Moreover, lung metastasis occurred in 100% (6 of 6) in Lenti‐miR‐186 & Lenti‐METTL3 group, which was significantly higher than that in Lenti‐miR‐186 group (1 of 6). No lung metastasis occurred in Lenti‐anti‐miR‐186 & sh‐METTL3 group (0 of 6), while in Lenti‐anti‐miR‐186 group, the incidence of lung metastasis was 50% (3 of 6; Figure 7H,I). To further confirm the metastasis results in a system that more accurately mimic the in vivo environment, an orthotopic HB transplantation model in C57BL/6 mice was established. Fluorescence images demonstrated that miR‐186 overexpression resulted in significantly decreased tumour size (Figure 7J,K). The orthotopic xenograft tumours in the Lenti‐miR‐186 group had drastically lower liver weight/body weight ratios (Figure 7L,M). These results indicate that miR‐186 acts as a potential tumour suppressor in HB via negatively regulating METTL3.

### 3.8 miR‐186/METTL3 axis regulates Wnt/β‐catenin signalling in human HB

**Figure 8:**
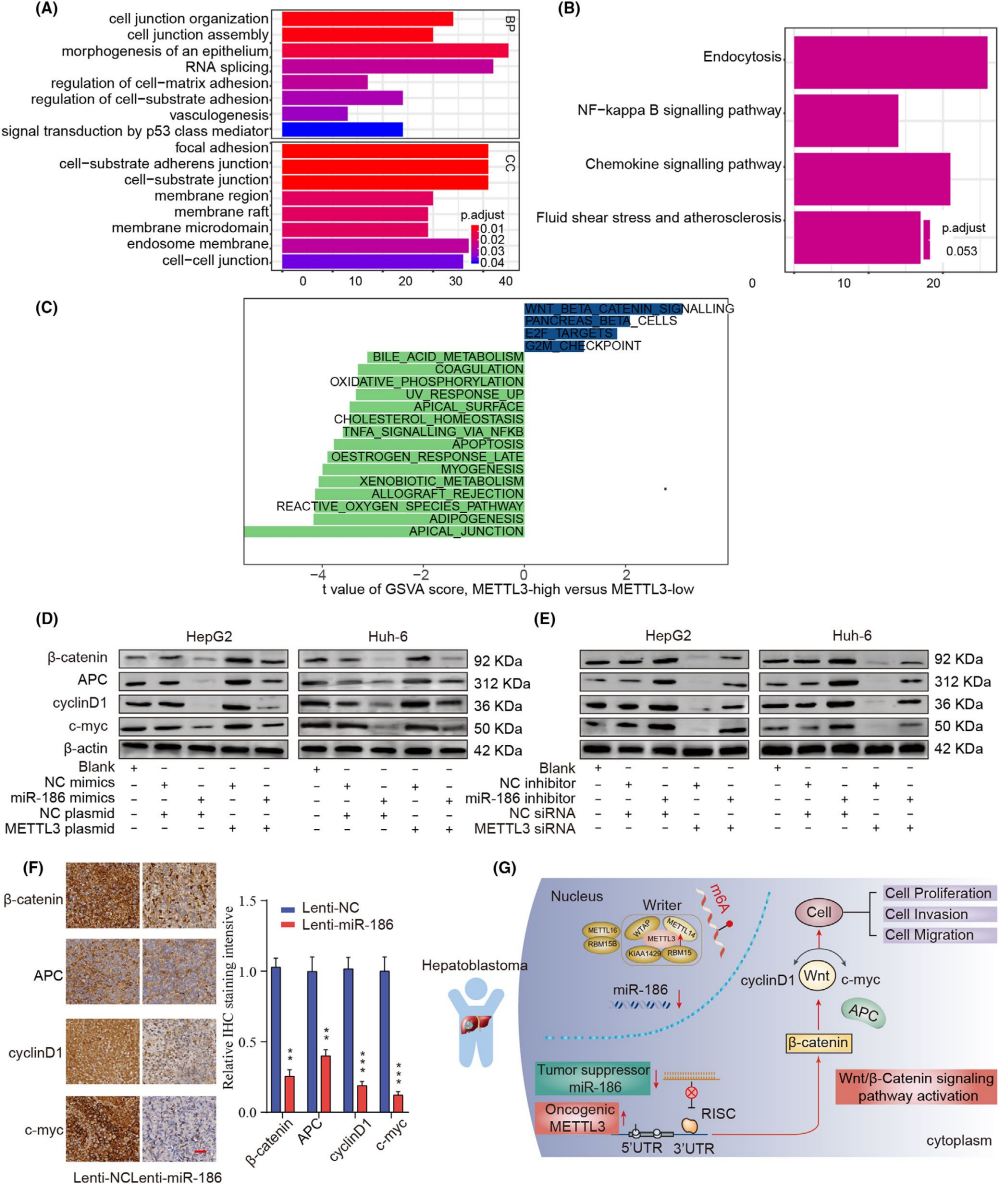
miR‐186/METTL3 axis regulates Wnt/β‐catenin signalling in human HB. A, GO and (B) KEGG analysis showed the enriched biological functions of differentially expressed gene profiles. C, GSVA showed the correlations between METTL3 mRNA levels and signalling pathways. D, E, The protein levels of Wnt/β‐catenin pathway‐related targets in HepG2 or HuH‐6 cells transfected with miR‐186 mimics, METTL3 plasmid, miR‐186 inhibitor, METTL3 siRNA or relative NC. F, Representative immunohistochemical staining images and relative expression levels of Wnt/β‐catenin pathway‐associated proteins in tumours from Lenti‐miR‐186 group and Lenti‐NC group. Scale bars, 200 μm. G, The proposed function model of miR‐186/METTL3 axis in HB

To explore the potential mechanisms related to the miR‐186/METTL3 in HB, we performed bioinformatics analysis based on GEO microarray (GSE75271). The results revealed that the differentially expressed gene profiles were enriched in multiple signal pathways or biological processes in Gene Ontology (GO) or Kyoto Encyclopedia of Genes and Genomes (KEGG) analysis, respectively (Figure 8A,B). In addition, Gene Set Variation Analysis (GSVA) revealed that Wnt/β‐catenin signalling pathway ranked the top 1 signalling associated with upregulated METTL3 expression (Figure 8C). Given the crucial role of Wnt/β‐catenin signalling in the regulation of tumour cell proliferation, migration and invasion,^25^ we chosen the Wnt/b‐catenin signalling for further validation. We demonstrated that the expression levels of Wnt/β‐catenin pathway‐associated proteins, such as β‐catenin, APC, cyclinD1 and c‐myc were dramatically increased in METTL3‐overexpressed HepG2 or HuH‐6 cells but were decreased in METTL3‐silenced cells (Figure 8D,E). However, the expression levels of proteins associated with Wnt/β‐catenin pathway were inversely correlated with miR‐186 expression (Figure 8D,E). Additionally, IHC staining in xenograft tumour of nude mice also showed decreased expression of Wnt/β‐catenin pathway‐associated proteins in the Lenti‐miR‐186 group (Figure 8F). Collectively, these data suggest that miR‐186/METTL3 axis might affect the biological behaviour of HB through Wnt/β‐catenin pathway.

## 4 DISCUSSION

Increasing number of studies has revealed that m6A plays crucial roles in human diseases. For example, m6A modification may lead to obesity,^26^ type 2 diabetes mellitus^27^ and infertility.^28^ However, the expression pattern and specific role of the m6A modification in human diseases have been largely unknown. Recently, emerging evidence has demonstrated that m6A‐related genes play essential roles in the mutagenesis and carcinogenesis of human cancers. METTL3 was shown to participate in hepatocellular carcinoma (HCC) progression via mRNA m6A modification.^13^ RBM15 and METTL14 were reported to play oncogenic roles in leukemogenesis.^29^ In addition, YTHDF1 was elucidated to be an oncogene in colorectal cancer.^30^ However, less is known about the critical role of m6A‐related molecules in HB. In this study, we investigated the expression profiles of m6A‐related molecules in HB at both the mRNA and protein levels. Through transcriptomic and proteomic analyses of m6A‐associated molecules in large HB cohorts, including RNA‐seq data from public GEO microarray platform and IHC staining results from the TMA ZZU cohort, we found that m6A‐related genes were frequently dysregulated in HB and that the upregulation of METTL3 expression was an independent survival risk factor for HB (Figures 1,2 and 3A).

For the first time, we reported the function of METTL3 in HB and illuminated the potential mechanisms. METTL3 was significantly upregulated in mRNA and protein levels and was associated with unpleasant prognosis in HB (Figure 3). Functional assays showed that knockdown of METTL3 significantly inhibited cell proliferative, migration and invasion of both HepG2 and HuH‐6 cells in vitro (Figure 4)*.* Consistent with our reports, recent study demonstrated that METTL3‐mediated m6A modification promoted liver cancer progression.^13^ Zhang Z et al reported that downregulation of METTL3 significantly impeded bladder cancer cell proliferation, and invasion in vitro and tumorigenicity in vivo.^31^ Cai X et al also showed that METTL3 promoted the progression of breast cancer by inhibiting tumour suppressor let‐7g.^32^ In summary, our study robustly demonstrated that METTL3 was significantly upregulated and functioned as an oncogene in human HB.

We corroborated that METTL3 was a directly functional target of miR‐186 (Figure 5). miR‐186 has been identified as a tumour suppressor and found to be downregulated in certain malignancies; for example, emerging studies have demonstrated that miR‐186 could suppress the growth and metastasis of bladder cancer and hepatocellular carcinoma.^33^, ^34^ In addition, Zhang, J.J et al validated that miR‐186 could play the inhibitory roles in the progression of cervical cancer.^35^ Huang T et al also showed miR‐186 could inhibit migration of non–small‐cell lung cancer.^36^ Consistent with these findings, our data revealed that downregulation of miR‐186 significantly stimulated malignant progression and associated with poor clinical outcomes in HB‐afflicted patients. In addition, overexpression of miR‐186 inhibits HB cell proliferation, migration and invasion in vitro, and inhibits HB tumour development and lung metastasis in vivo (Figures 6 and 7), which could be partially reversed by the overexpression of METTL3. These results suggest that miR‐186 functions as tumour suppressor in HB via targeting METTL3.

For mechanism exploration, we identified an association between miR‐186/METTL3 axis and Wnt/β‐catenin pathway through the GSVA (Figure 8). Dysregulation of Wnt/β‐catenin signalling occurs in many types of malignant cancers,^37^, ^38^, ^39^ including HB. C. Armengol et al reported the Wnt/β‐catenin pathway played a key role in liver development in hepatoblastoma model.^40^ Another report demonstrated that Wnt/β‐catenin pathway was associated with novel advances of molecular pathogenesis of hepatoblastoma.^41^ Ueda Y et al also indicated that Wnt/β‐catenin signalling positively correlated with chemosensitivity and surgical resectability of hepatoblastoma.^42^ Our data showed that overexpression of METTL3 was positively correlated with the expression of Wnt/β‐catenin pathway‐associated proteins by Western blot assay and miR‐186 was found to be involved in the regulation of METTL3 and Wnt/β‐catenin signalling pathway. However, due to the complexity of tumour microenvironment, it is possible that multiple signalling pathways are involved in HB development and progression, such as NF‐kB and p53 signalling pathways, we would like to further explore the function of these signalling pathways in our future studies. Taken together, we propose that miR‐186 interacting with METTL3 contributes to the progression of HB via activating Wnt/β‐catenin signalling pathway (Figure 8G).

Nevertheless, the study still has a few shortcomings. First, one gene could be targeted and regulated by multiple molecules; other miRNAs might also contribute to the HB progression through regulating METTL3. Second, we revealed that the Wnt/β‐catenin signalling pathway was involved in HB development regulated by oncogenic METTL3. However, METTL3 has recently been reported to play crucial roles through m6A epitranscriptional modification in several common cancers.^43^, ^44^ Whether METTL3 affects the progression of HB through a m6A modification pattern remains a subject for further study.

## 5 CONCLUSIONS

In summary, M6A‐related genes were frequently dysregulated in HB. METTL3, an m6A‐associated RNA methyltransferase is highly expressed and functions as an oncogene in HB. We demonstrate that miR‐186 could regulate METTL3 expression in progression of HB via Wnt/β‐catenin signalling, suggesting the miR‐186/METTL3 axis may be used as an effective therapeutic and prognostic biomarker for HB‐afflicted patients.

## CONFLICT OF INTERESTS

The authors confirm that there are no conflicts of interests.

## AUTHORS’ CONTRIBUTIONS

XCC, ZFW and RRS performed all the experimental work. JHL, ZGR and DDZ participated in data analysis. JMZ, WZ, JHL and DDZ designed and conducted the animal experiment. YZF and DZ conceived and participated in the design of the study. The manuscript was written by XCC, ZFW and RRS. All authors read and approved the final manuscript.

## Data Availability

The data that support the findings of this study are available from the corresponding author upon reasonable request.
