# Supplementary figures and images for: Cross talk between RNA N6‐methyladenosine methyltransferase‐like 3 and miR‐186 regulates hepatoblastoma progression through Wnt/β‐catenin signalling pathway
Source: Cell Prolif. 2020 Jan 22;53(3):e12768. doi: 10.1111/cpr.12768 (PMC7106953; doi:10.1111/cpr.12768)

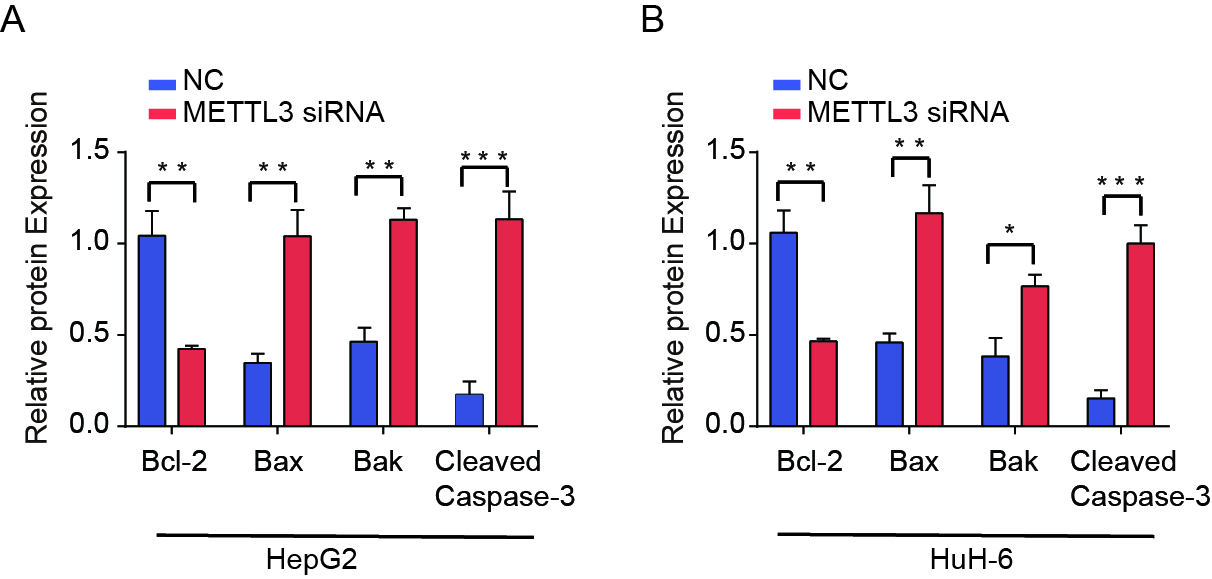

Supplement: Supplementary file 1 [file CPR-53-e12768-s001.tif]

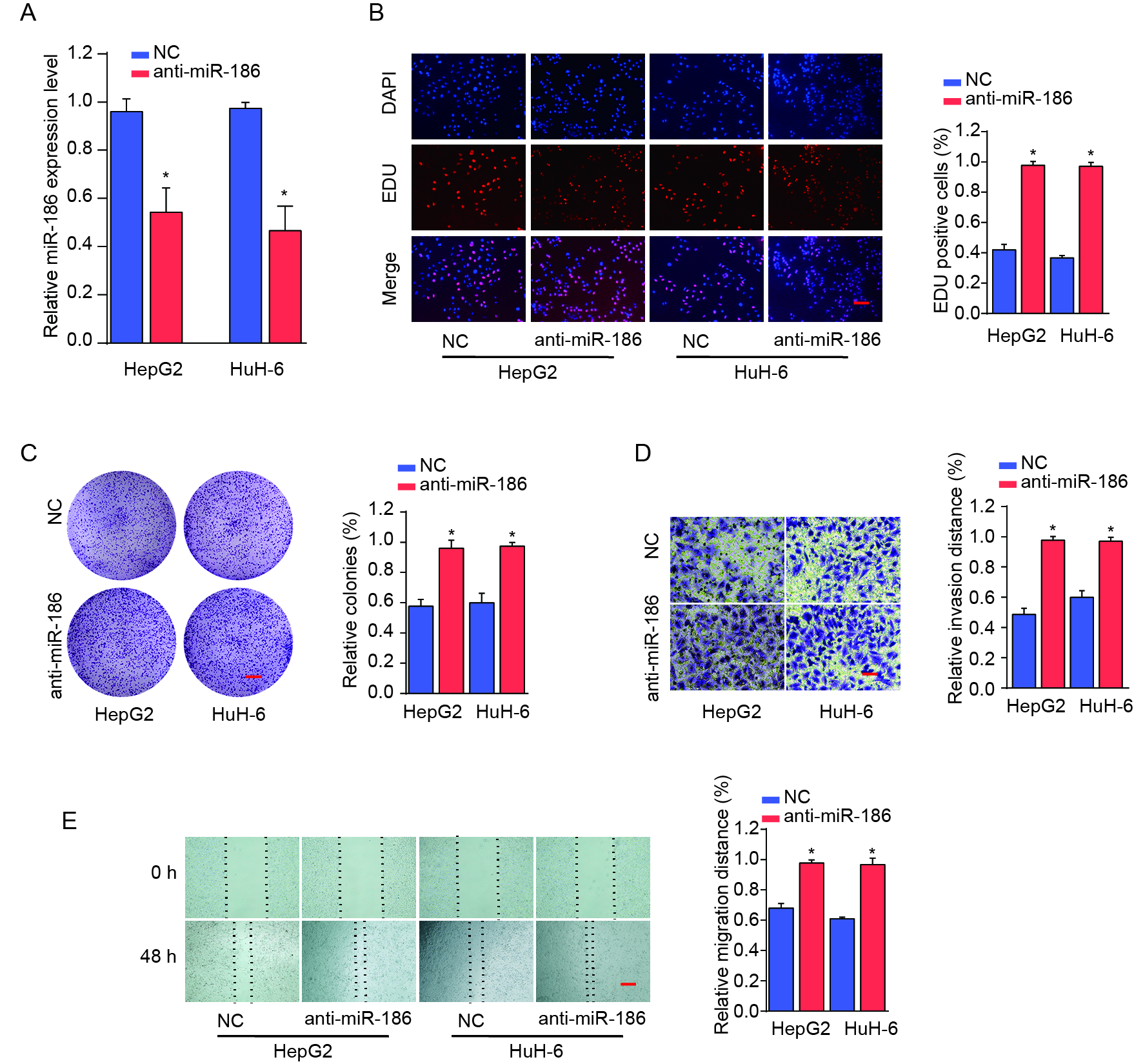

Supplement: Supplementary file 2 [file CPR-53-e12768-s002.tif]

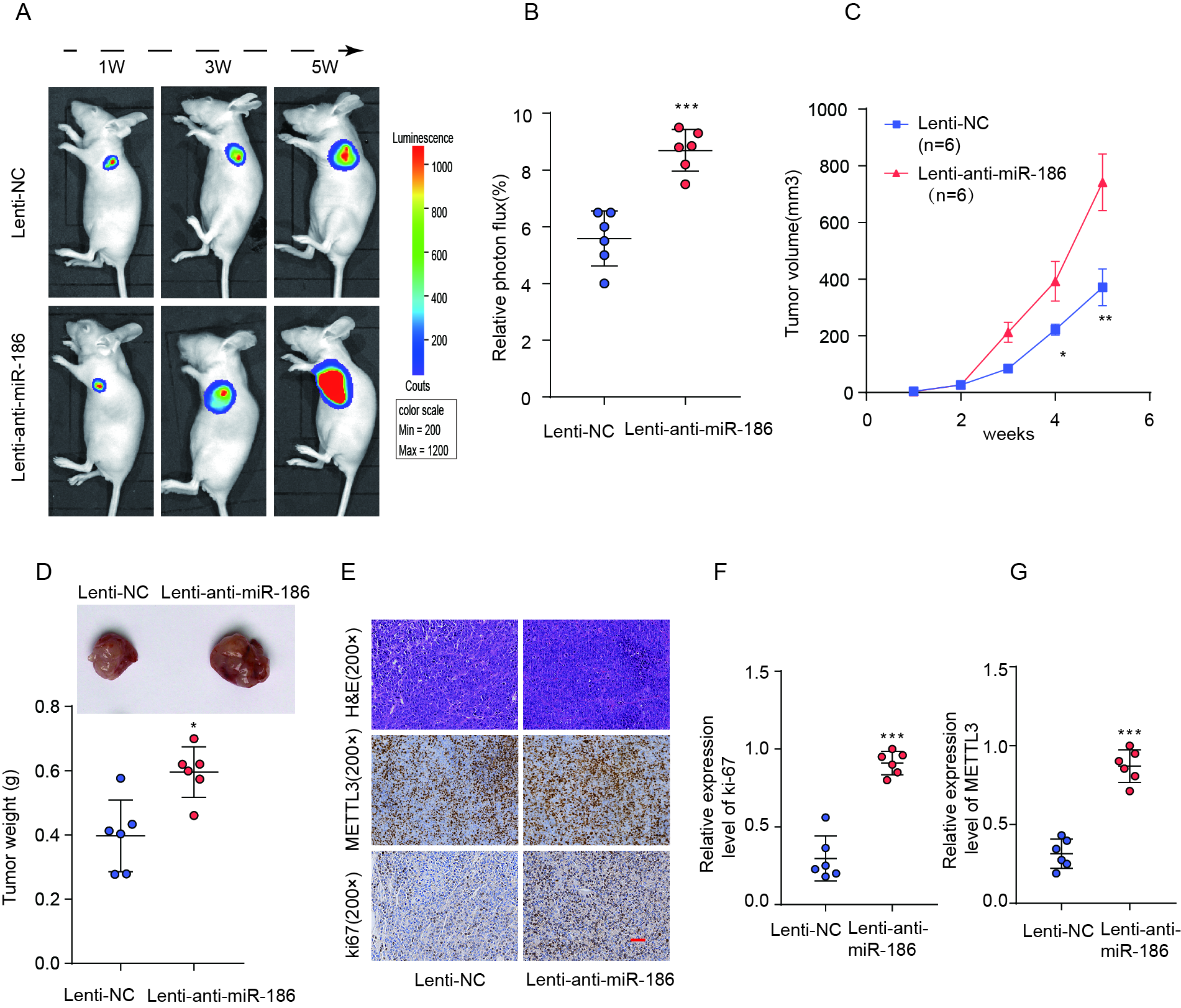

Supplement: Supplementary file 3 [file CPR-53-e12768-s003.tif]
