# Supplementary material for: Cross talk between RNA N6‐methyladenosine methyltransferase‐like 3 and miR‐186 regulates hepatoblastoma progression through Wnt/β‐catenin signalling pathway
Source: Cell Prolif. 2020 Jan 22;53(3):e12768. doi: 10.1111/cpr.12768 (PMC7106953; doi:10.1111/cpr.12768)
Supplement: Supplementary file 4 [file CPR-53-e12768-s004.docx]

**Table S1. GEO information used in this study**

| GEO ID | Platforms | Non-tumor | Tumor | Year | Country |
| --- | --- | --- | --- | --- | --- |
| GSE75271 | Affymetrix mRNA microarray | 5 | 50 | 2016 | USA |
| GSE75283 | Agilent miRNA microarray | 8 | 57 | 2017 | USA |
| Total |  | **13** | **107** |  |  |
