# Supplementary material for: Cross talk between RNA N6‐methyladenosine methyltransferase‐like 3 and miR‐186 regulates hepatoblastoma progression through Wnt/β‐catenin signalling pathway
Source: Cell Prolif. 2020 Jan 22;53(3):e12768. doi: 10.1111/cpr.12768 (PMC7106953; doi:10.1111/cpr.12768)
Supplement: Supplementary file 5 [file CPR-53-e12768-s005.docx]

**Table S2. Association of METTL3 expression and clinicopathological features**

| Clinicopathological features | | No. of cases (%) | METTL3 | | *P*-value |
| --- | --- | --- | --- | --- | --- |
|  |  |  | Low  (n=29) | High  (n=41) |  |
| Age (years) | ≤Median | 35 | 12 | 23 | 0.332 |
|  | >Median | 35 | 17 | 18 |  |
| Gender | Male | 44 | 14 | 30 | 0.135 |
|  | Female | 26 | 15 | 11 |  |
| Tumor size | ≤10cm | 36 | 11 | 25 | 0.088 |
|  | >10cm | 34 | 18 | 16 |  |
| Vascular invasion | Absent | 55 | 27 | 28 | **0.017** |
|  | Present | 15 | 2 | 13 |  |
| AFP | ≤100 ng/ml | 36 | 19 | 17 | 0.561 |
|  | >100 ng/ml | 34 | 10 | 24 |  |
| Histologic type | Epitheliated | 52 | 23 | 29 | 0.580 |
|  | Mixed | 18 | 6 | 12 |  |
| Metastasis | Absent | 52 | 26 | 26 | **0.015** |
|  | Present | 18 | 3 | 15 |  |
| Recurrence | Absent | 57 | 27 | 30 | **0.010** |
|  | Present | 13 | 1 | 12 |  |
| COG stage | Stage I-II | 41 | 22 | 19 | **0.016** |
|  | Stage III-IV | 29 | 7 | 22 |  |
