# Supplementary material for: Cross talk between RNA N6‐methyladenosine methyltransferase‐like 3 and miR‐186 regulates hepatoblastoma progression through Wnt/β‐catenin signalling pathway
Source: Cell Prolif. 2020 Jan 22;53(3):e12768. doi: 10.1111/cpr.12768 (PMC7106953; doi:10.1111/cpr.12768)
Supplement: Supplementary file 6 [file CPR-53-e12768-s006.docx]

**Table S3. Cell lines used in this study**

| **Cell lines** | **Cell type** | **Source** | **Country** |
| --- | --- | --- | --- |
| HepG2 | Hepatoblastoma cell | Cell Bank of the Chinese  Academy of Science | China |
| HuH-6 | Hepatoblastoma cell | Cell Bank of the Chinese  Academy of Science | China |
| Chang liver | Normal liver cell | ATCC | USA |
| L02 | Normal liver cell | SIBCB | China |
| HEK293 | Embryonic kidney cell | Cell Bank of the Chinese  Academy of Science | China |
| HCCLM9 | Hepatocellular carcinoma cell | Cell Bank of the Chinese  Academy of Science | China |
| Hepa1-6 | Hepatocellular carcinoma cell | Cell Bank of the Chinese  Academy of Science | China |
