# Supplementary material for: Cross talk between RNA N6‐methyladenosine methyltransferase‐like 3 and miR‐186 regulates hepatoblastoma progression through Wnt/β‐catenin signalling pathway
Source: Cell Prolif. 2020 Jan 22;53(3):e12768. doi: 10.1111/cpr.12768 (PMC7106953; doi:10.1111/cpr.12768)
Supplement: Supplementary file 7 [file CPR-53-e12768-s007.docx]

**Table S4. Information on antibodies used in this study**

| **Antibody** | **WB** | **IHC** | **Specificity** | **Company** |
| --- | --- | --- | --- | --- |
| β-actin | 1:5000 | / | Mouse monoclonal | Proteintech Group, China |
| METTL3 | 1:2000 | 1:100 | Rabbit Polyclonal | Proteintech Group, China |
| Ki-67 | / | 1:500 | Rabbit Polyclonal | Proteintech Group, China |
| β-catenin | 1:1000 | / | Rabbit Polyclonal | Proteintech Group Chicago, USA |
| APC | 1:1000 | / | Rabbit Polyclonal | Proteintech Group Chicago, USA |
| cyclinD1 | 1:1000 | / | Rabbit Polyclonal | Proteintech Group Chicago, USA |
| c-myc | 1:1000 | / | Rabbit Polyclonal | Proteintech Group Chicago, USA |
