# Supplementary material for: Cross talk between RNA N6‐methyladenosine methyltransferase‐like 3 and miR‐186 regulates hepatoblastoma progression through Wnt/β‐catenin signalling pathway
Source: Cell Prolif. 2020 Jan 22;53(3):e12768. doi: 10.1111/cpr.12768 (PMC7106953; doi:10.1111/cpr.12768)
Supplement: Supplementary file 8 [file CPR-53-e12768-s008.docx]

**Table S5. Univariate and multivariate analyses of overall survival of hepatoblastoma**

| Clinicopathological features | | Univariate analyses | | *P*-value | Multivariate analyses | | *P*-value |
| --- | --- | --- | --- | --- | --- | --- | --- |
|  |  | HR | 95% (CI) |  | HR | 95% (CI) |  |
| Age (years) | <Median | 1.027 | 0.436-2.420 | 0.951 |  |  |  |
|  | >Median |  |  |  |  |  |  |
| Gender | Male | 0.882 | 0.365-2.130 | 0.781 |  |  |  |
|  | Female |  |  |  |  |  |  |
| Tumor size | ≤10cm | 1.674 | 0.693-4.044 | 0.252 |  |  |  |
|  | >10cm |  |  |  |  |  |  |
| AFP | ≤100 ng/ml | 0.972 | 0.225-4.192 | 0.969 |  |  |  |
|  | >100 ng/ml |  |  |  |  |  |  |
| Histologic type | Epitheliated type | 0.868 | 0.317-2.378 | 0.783 |  |  |  |
|  | Mixed |  |  |  |  |  |  |
| Vascular invasion | Absent | 2.863 | 1.136-7.217 | **0.026** | 1.000 |  |  |
|  | Present |  |  |  | 7.772 | 2.417-24.995 | **0.001** |
| metastasis | Absent | 7.841 | 3.133-19.623 | **0.000** | 1.000 |  |  |
|  | Present |  |  |  | 2.052 | 0.678-6.209 | **0.203** |
| Recurrence | Absent | 7.171 | 2.989-17.204 | **0.000** | 1.000 |  |  |
|  | Present |  |  |  | 4.625 | 1.627-13.145 | **0.004** |
| COG stage | Stage I-II | 2.699 | 1.103-6.457 | **0.003** | 1.000 |  |  |
|  | Stage III-IV |  |  |  | 2.909 | 1.112-7.613 | **0.030** |
| METTL3 | Low | 5.157 | 1.517-17.527 | **0.009** | 1.000 |  |  |
|  | High |  |  |  | 5.265 | 1.168-23.730 | **0.031** |
| YTHDF2 | Low | 2.418 | 1.025-5.704 | **0.044** | 1.000 |  |  |
|  | High |  |  |  | 2.102 | 0.773-5.718 | 0.146 |
| FTO | Low | 3.696 | 1.088-12.550 | **0.036** | 1.000 |  |  |
|  | High |  |  |  | 1.532 | 0.351-6.680 | 0.570 |
| WTAP | Low | 0.461 | 0.136-1.568 | 0.215 |  |  |  |
|  | High |  |  |  |  |  |  |
| KIAA1429 | Low | 0.552 | 0.213-1.426 | 0.220 |  |  |  |
|  | High |  |  |  |  |  |  |
| RBM15 | Low | 0.804 | 0.269-2.402 | 0.697 |  |  |  |
|  | High |  |  |  |  |  |  |
| RBM15B | Low | 0.691 | 0.291-1.642 | 0.403 |  |  |  |
|  | High |  |  |  |  |  |  |
| METTL14 | Low | 1.657 | 0.556-4.936 | 0.364 |  |  |  |
|  | High |  |  |  |  |  |  |
| METTL16 | Low | 0.516 | 0.189-1.410 | 0.197 |  |  |  |
|  | High |  |  |  |  |  |  |
| HNRNPC | Low | 0.512 | 0.119-2.197 | 0.367 |  |  |  |
|  | High |  |  |  |  |  |  |
| HNRNPA2B1 | Low | 0.446 | 0.171-1.162 | 0.099 |  |  |  |
|  | High |  |  |  |  |  |  |
| YTHDF1 | Low | 0.561 | 0.226-1.390 | 0.212 |  |  |  |
|  | High |  |  |  |  |  |  |
| YTHDF3 | Low | 0.381 | 0.128-1.132 | 0.381 |  |  |  |
|  | High |  |  |  |  |  |  |
| YTHDC1 | Low | 0.552 | 0.162-1.880 | 0.342 |  |  |  |
|  | High |  |  |  |  |  |  |
| ALKBH5 | Low | 0.257 | 0.661-1.705 | 0.392 |  |  |  |
|  | High |  |  |  |  |  |  |
