# Supplementary material for: Cross talk between RNA N6‐methyladenosine methyltransferase‐like 3 and miR‐186 regulates hepatoblastoma progression through Wnt/β‐catenin signalling pathway
Source: Cell Prolif. 2020 Jan 22;53(3):e12768. doi: 10.1111/cpr.12768 (PMC7106953; doi:10.1111/cpr.12768)
Supplement: Supplementary file 9 [file CPR-53-e12768-s009.docx]

**Figure. S1 Downregulation of METTL3 promotes HB cell apoptosis.** (**A, B**) Western blot analysis of Bcl-2, Bax, Bak and cleaved caspase-3 proteins in HepG2 and HuH-6 cells. **p*< 0.05, ***p*< 0.01, ****p*< 0.001.

**Figure. S2 Downregulation of miR-186 promotes HB cell proliferation, migration and invasion.** HepG2 or HuH-6 cells were transfected with NC or anti-miR-186. (**A**) The expression levels of miR-186 in HB cell lines were determined by qRT-PCR. (**B**) EDU staining (scale bars, 50μm) and (**C**) colony formation assays (scale bars, 8mm) were performed to determine the cell proliferation activity. (**D**) Transwell assay was conducted to assess cell invasion capacity. Scale bars, 50μm. (**E**) Wound-healing assay was conducted to assess the cell migration capacity. Scale bars, 500μm. **p <*0.05.

**Figure. S3 Downregulation of miR-186 promotes tumour growth *in vivo*.** (**A**) Representative image of Luciferase signal emission in Lenti-anti-miR-186 group and Lenti-NC group. (**B**) Relative photon flux in Lenti-anti-miR-186 group and Lenti-NC group were quantified and analysed using the IVIS imaging system 5 weeks after implantation. (**C**) Growth curves of tumour volumes in xenografts of nude mice were determined based on tumour volume measured every week. (**D**) Representative images of tumour and quantitative analysis of xenograft tumour weight in Lenti-NC and Lenti-anti-miR-186 groups. (**E-G**) Representative immunohistochemical staining images and relative expression levels of ki-67 and METTL3 in tumours from Lenti-anti-miR-186 group and Lenti-NC group. Scale bars, 200μm. **p <*0.05. ***p* < 0.01, ****p*< 0.001.
